# Improved Performance of Polylactic Acid Biocomposites at High Lignin Loadings through Glycidyl Methacrylate Grafting of Melt-Flowable Organosolv Lignin

**DOI:** 10.1021/acsomega.4c05212

**Published:** 2024-08-06

**Authors:** Shallal Alshammari, Amir Ameli

**Affiliations:** Department of Plastics Engineering, University of Massachusetts Lowell, 1 University Ave, Lowell, Massachusetts 01854, United States

## Abstract

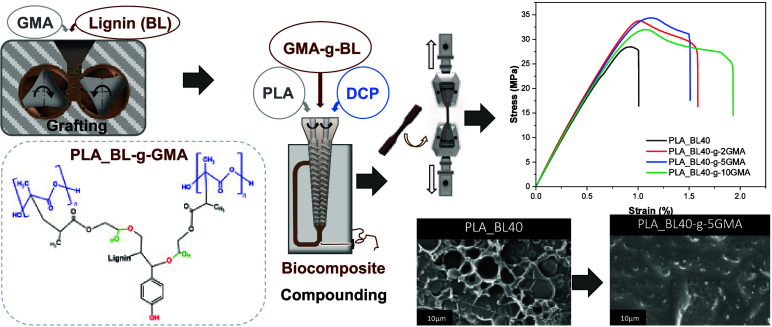

Glycidyl methacrylate (GMA) was grafted onto a melt-flowable organosolv lignin, called bioleum (BL), using a melt mixing process. Then, up to 40 wt % of BL-*g*-GMA was blended with polylactic acid (PLA) in the presence of dicumyl peroxide as a free radical initiator utilizing a melt extrusion method. Fourier transform infrared spectroscopy (FTIR), thermogravimetric analysis, differential scanning calorimetry, scanning electron microscopy, and tensile testing were performed to characterize the biocomposites’ performance. The FTIR results revealed a successful grafting of GMA onto BL. Overall, BL and PLA compatibility increased significantly with the grafting and resulted in decreased domain size of BL-*g*-GMA and thus enhanced all the tensile properties (strength, modulus, and elongation at break) at BL loadings as high as 40 wt %. For instance, in the biocomposites containing 30 wt % BL, the GMA grafting increased the tensile strength by 23%. The presence of BL and BL-*g*-GMA hindered PLA’s crystallization even when it was cooled at a rate of 1 °C/min. However, the composites with BL-*g*-GMA showed a crystallinity value comparable to that of PLA during isothermal crystallization, but with a slower crystallization rate. This work reveals a facile and scalable method that can be adopted to enhance the performance of lignin-based biocomposites.

## 1 Introduction

Biobased and biodegradable plastics are increasingly essential in modern society due to their environmentally friendly characteristics, such as environmental sustainability, reduced landfill waste, and lower greenhouse gas emissions.^1^ Among them, poly(lactic acid) (PLA) is a very promising alternative to petroleum-based plastics due to its excellent mechanical and chemical properties. However, PLA production can be more expensive compared to traditional petroleum-based plastics due to the higher costs associated with the extraction and refining of raw materials as well as the processing techniques involved. Moreover, PLA, being an aliphatic polyester, has limited functional groups, which narrows down its interactions with other molecules, limiting its applicability in some fields such as medicine and packaging.^2,3^ Therefore, several studies have been conducted on the compounds of PLA with biobased materials, such as cellulose and lignin, in order to enhance its performance or reduce its costs.^4,5^

Since lignin is an inexpensive and abundant natural aromatic polymer, its potential use as a component in bioplastic composites, such as mixing with PLA, has been gaining tremendous attention in both industry and academia.^6^ However, the polarity of lignin molecules causes strong self-interactions and makes its efficient dispersion and blending with other polymers challenging. Several types of unmodified lignin, such as kraft, lignosulfonate, organosolv, and soda, have been incorporated into PLA in the last few decades, and the resulting composites show poor dispersion and interfacial adhesion which results in reduced mechanical properties, compared to neat PLA.^7−13^ In most reported cases, at relatively high lignin loading (15–40 wt %) of unmodified commercial lignin, the tensile strength drops significantly by more than 50%. Gordobil et al.^8^ reported that the addition of 20 wt % alkaline and organosolv lignin reduced the tensile strength by 83 and 54%, respectively. Obielodan et al.^14^ also found that the addition of 30 and 40 wt % organosolv lignin to the PLA matrix reduced its tensile strength by 61 and 68%, respectively, compared to that of PLA. They also reported that the reduction in mechanical strength is attributed to the insufficient dispersion of lignin and the weak interfacial interactions between PLA and lignin. This phenomenon is due to the formation of self-agglomerates by strong intermolecular hydrogen bonding of unmodified lignin during polymer processing, leading to a decrease in the compatibility of lignin in the PLA matrix.^15^

Therefore, lignin is often modified chemically using different modification processes, such as esterification,^16−18^ acetylation^9,19,20^ and graft copolymerization,^21−28^ in order to improve its dispersion and interfacial adhesion in PLA. Hong et al.^17^ esterified organosolv lignin with maleic anhydride, then melt blended with PLA to prepare composites. It was found that esterification decreased the hydroxyl groups in lignin and improved the interfacial compatibility of the composite, which resulted in more than 2-fold improvement in the tensile strength compared to the unmodified lignin. Kim et al.^19^ blended virgin lignin and acetylated lignin with PLA to prepare composites. It was observed that the acetylation process caused a uniform dispersion with a significant reduction in the lignin particle size in the PLA matrix, due to a decrease in hydrogen bonding strength between lignin molecules. They reported that the acetylation process decreased the average lignin particle diameter by around 34, 45, and 43% in the PLA composites having 1, 5, and 10 wt % lignin, respectively.

Among these chemical modification techniques, graft copolymerization has proven to be one of the most effective routes. It is considered as a promising method for preparing functional materials with new properties.^29^ With graft copolymerization, PLA or lignin can be functionalized by introducing various other monomers or oligomers that provide suitable functional groups, enabling grafting onto other polymers. Ge et al.^21^ grafted maleic anhydride (MA) directly onto PLA in the presence of dibenzoyl peroxide (BPO) as a free radical initiator. During the mixing of PLA and lignin, grafted MA reacted with the hydroxyl groups in lignin to form ester bonds. This modification resulted in an improvement in the compatibility of PLA and lignin. The tensile strength and elongation at break were improved significantly and reached the highest in the composite with 4 wt % lignin. They increased from 59.9 MPa and 3.8% in the unmodified composite to 71.6 MPa and 5.0% in the sample with MA-BPO. This can be ascribed to the strong interaction between grafted PLA and lignin and the high toughness of MA formed in the composites. Wang et al.^28^ prepared polylactide-*graft*-glycidyl methacrylate (PLA-*g*-GMA) to be used as a compatibilizer to enhance the interfacial adhesion and mechanical properties of PLA/lignin composites. The PLA-*g*-GMA significantly increased the tensile strength in the PLA composite with 3 wt % lignin, resulting in 33% higher tensile strength than the neat PLA. The reason for this was that the epoxy group in PLA-*g*-GMA was able to react with the hydroxyl group on the lignin surface and also with the hydroxyl and carboxyl end groups of PLA, forming in situ chemical bonds that improved the PLA/lignin interfacial adhesion. It is however noted that these works studied only the low loading of lignin up to 4 and 5 wt %, respectively, and used additional preprocessing steps.

Generally, chemical modification methods require preprocessing steps involving solvents, and they are usually time-consuming. Furthermore, the poor rheological characteristics of lignin also often limit the amount of lignin that may be effectively incorporated in biocomposites during melt processing. Recently, a new melt-flowable organosolv lignin, named bioleum (BL), has been developed which can address the flowability issues.^30^ The melt-flowable characteristic of BL has made the lignin’s chemical modification possible during reactive melt processing in a fast, solvent-free, and continuous process with no need for using plasticizers. Also, as reported in our previous study, PLA/BL mixtures exhibited melt extrusion viability at high loadings of BL, up to 40 wt %, due to the favorable rheological behavior of BL and the resultant composite melt.^31^

**Figure 1:**
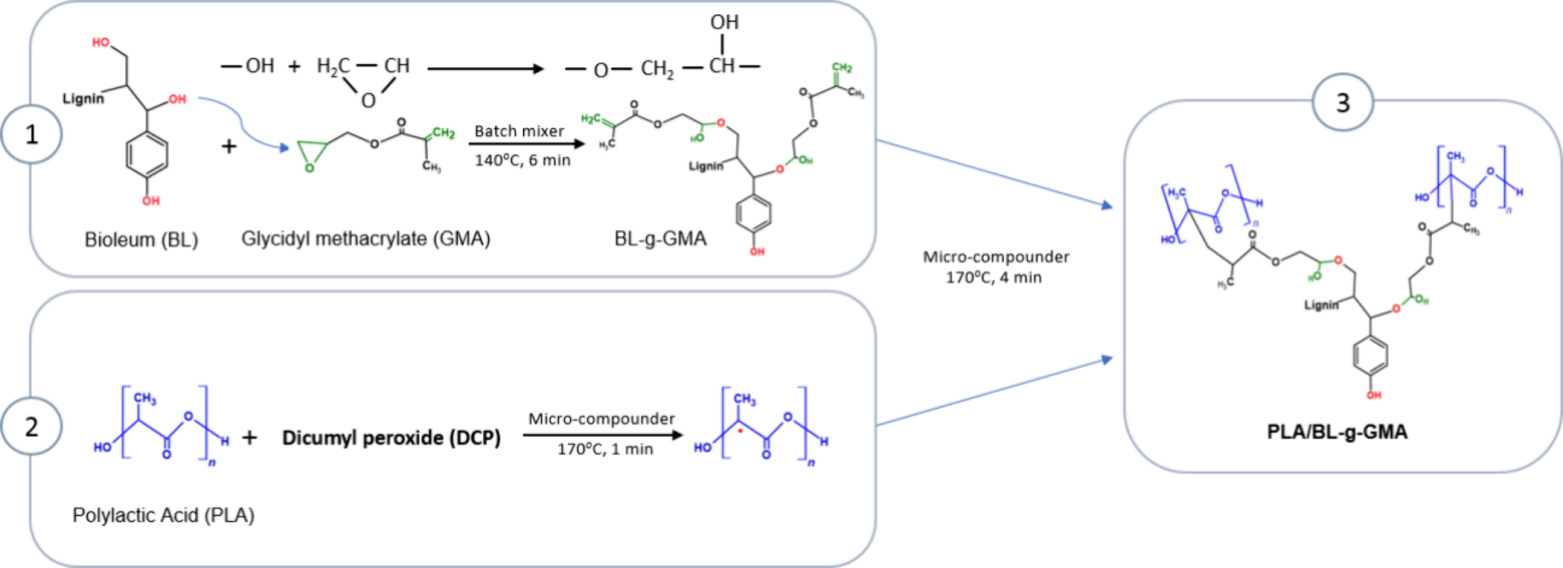
Scheme of the possible reaction mechanisms among BL, GMA, and PLA.

In this study, a solvent-free melt mixing process was first used to graft different ratios (2, 5, and 10 wt %) of glycidyl methacrylate (GMA) onto BL. Bioleum-grafted-glycidyl methacrylate (BL-*g*-GMA), up to 40 wt %, was then blended with PLA in the presence of 0.1 wt % DCP as a free radical initiator, using a melt extrusion method. GMA is a highly reactive bifunctional monomer that contains both epoxy and acrylic acid groups.^28^ The epoxy groups can react with the hydroxyl group in lignin to reduce the surface polarity of lignin, making it more compatible with the PLA matrix. At the same time, the acrylic groups can graft GMA radicals to PLA chains, which can further enhance the composites’ mechanical properties. Figure 1 shows the scheme of possible reaction mechanisms among BL, GMA, and PLA. FTIR, thermogravimetric analysis (TGA), differential scanning calorimetry (DSC), scanning electron microscopy (SEM), and tensile testing were performed to characterize the performance of the resultant biocomposites.

## 2 Materials and Methods

### 2.1 Materials

PLA (Ingeo Biopolymer 2003D, with a melt flow rate of 6 g/10 min) was purchased from NatureWorks LLC. Bioleum (a melt flowable organosolv biolignin extracted from hardwood) was obtained in powder form from Comstock Inc. (Virginia City, NV, USA). Glycidyl methacrylate (GMA), with a density of 1.042 g/mL at 25 °C, and dicumyl peroxide (DCP), with a density of 1.56 g/mL at 25 °C, were purchased from Sigma-Aldrich and used as received.

### 2.2 Preparation of BL-*g*-GMA

First, BL was ground to reduce the particle size and then dried in a temperature-controlled oven at 70 °C for 24 h. The dried BL powder was then mixed with various ratios of GMA, i.e., 2, 5, and 10 wt % with respect to the weight of BL and GMA, using a Brabender batch mixer with sigmoidal mixing elements (C.W. Brabender Instruments, South Hackensack, NJ) at 140 °C for 6 min using a rotor speed of 60 rpm. These three different grafting concentrations of GMA were chosen to cover a relatively wide range of grafting levels and their impact on various properties, which can then later be used toward the optimization of the formulation. In this process, the GMA monomers reacted with lignin under the influence of heat and shear, forming covalent bonds between the epoxy group in GMA and the hydroxyl group in the lignin structure. Afterward, the mixture was ground and dried in an oven at 40 °C for 2 days to minimize the hydrolysis of PLA during the extrusion process. To verify the grafting between GMA and BL, the processed BL-*g*-GMA was also washed several times with ethanol and distilled water through filter paper under vacuum to remove any unreacted GMA. Both washed and unwashed BL-*g*-GMA samples were tested using Fourier transform infrared spectroscopy (FTIR) as detailed in Section 3.1. The samples were washed to ensure that the increase in the carbonyl group peak’s intensity at 1710 cm^–1^, compared to the Neat BL sample, was due to the reaction between GMA and BL, and not due to the presence of unreacted GMA. It is noted that for the preparation of PLA/BL-*g*-GMA samples, only unwashed BL-*g*-GMA was used.

### 2.3 Preparation of PLA_BL and PLA_BL-*g*-GMA Biocomposites

PLA was dried using a temperature-controlled oven at 55 °C for 24 h. Dried PLA pellets were then mixed with BL or BL-*g*-GMA in the required proportions and fed into the twin-screw extruder. Compounding of PLA_BL and PLA_BL-*g*-GMA was performed using an Xplore HT15 microcompounder. For all of the biocomposites, the rotational speed of the screw and the barrel temperature were set to 100 rpm and 170 °C, respectively. The residence time was set such that the total time of material in the barrel, including feeding, was around 5 min. Table 1 lists the codes and formulations of the prepared biocomposites. The extruded strands were then ground and used to prepare type-V dog-bone tensile specimens (ASTM D638). The tensile specimens were made in a hot press (Carver 4394 Auto Series Plus). The applied prepressure during material softening was about 1000 psi for the first 3 min, and it was increased to 40,000 psi during shaping in the last 1 min. The hot press temperature was set at 170 °C for both the platens.

**Table 1:** Composition of the Biocomposites^a^

sample code	PLA (%)	BL (%)	BL-*g*-2%GMA (%)	BL-*g*-5%GMA (%)	BL-*g*-10%GMA (%)	DCP (%)
neat PLA	100					
PLA_BL10	90	10				
PLA_BL10-*g*-2GMA	90		10			0.1
PLA_BL10-*g*-5GMA	90			10		0.1
PLA_BL10-*g*-10GMA	90				10	0.1
PLA_BL20	80	20				
PLA_BL20-*g*-2GMA	80		20			0.1
PLA_BL20-*g*-5GMA	80			20		0.1
PLA_BL20-*g*-10GMA	80				20	0.1
PLA_BL30	70	30				
PLA_BL30-*g*-2GMA	70		30			0.1
PLA_BL30-*g*-5GMA	70			30		0.1
PLA_BL30-*g*-10GMA	70				30	0.1
PLA_BL40	60	40				
PLA_BL40-*g*-2GMA	60		40			0.1
PLA_BL40-*g*-5GMA	60			40		0.1
PLA_BL40-*g*-10GMA	60				40	0.1

a The sample code consists of the utilized materials, i.e., polylactic acid (PLA), bioleum (BL), and glycidyl methacrylate (GMA). The numbers following BL show the lignin content in total composite, and the numbers preceding GMA show the GMA content with respect to GMA and BL mass. Dicumyl peroxide was fixed at 0.1% of the total mass.

## 3 Characterizations

### 3.1 Fourier Transform Infrared (FTIR) Spectroscopy

A Nicolet iS50 instrument from Thermo Fisher Scientific (Waltham, MA, USA) was used in attenuated total reflection (ATR) mode to conduct the FTIR spectroscopy of the samples. The analysis was conducted in the wavelength range of 500 to 4000 cm^–1^. 64 scans with a resolution of 4 cm^–1^ were applied to each sample.

### 3.2 Rheological Analysis

An ARES G2 TA rheometer equipped with a parallel plate module of 25 mm diameter was used to analyze the rheological behavior. A frequency sweep from 0.01 to 100 rad/s with a strain fixed at 1% was used to analyze the complex viscosity of PLA, BL, PLA_BL20, and PLA_BL20-grafted to 2, 5, and 10% GMA. The frequency sweep tests were performed with a 2 mm gap (sample thickness) at 170 °C. Three repetitions were performed for each sample, and the mean values were reported.

### 3.3 Thermogravimetric Analysis (TGA)

A Mettler Toledo thermogravimetric analyzer 2 was used to perform the TGA tests. The samples (10–15 mg each) were heated from 50 to 600 °C at a heating rate of 20 °C/min under a nitrogen environment. The TGA data was analyzed using STARe software. The weight loss rate with time (DTG) was obtained by using the derivative of the thermogravimetric data. The onset, maximum, and endset decomposition temperatures denoted as *T*_5%_, *T*_max_, and *T*_endset_ as well as the residual weight at 600 °C were obtained and reported for each case.

### 3.4 Differential Scanning Calorimetry (DSC)

DSC was performed using a Mettler Toledo DSC 3+, operated under a nitrogen atmosphere at a 20 mL/min flow rate. PLA, BL, PLA_BL, and PLA_BL-*g*-GMA biocomposite samples (5–10 mg each) were heated from 25 to 200 °C at a heating rate of 10 °C/min (1) and kept at 200 °C for 5 min to remove any thermal history (2). The samples were then cooled to 25 °C at different cooling rates of 1, 2.5, and 5 °C/min (3). The second heating was then applied under the same conditions as the first heating (4).

For isothermal crystallization kinetics, the sample PLA_BL20 was heated from 25 to 200 °C at a heating rate of 10 °C/min (1) and kept at 200 °C for 5 min (2). The sample was then cooled at 60 °C/min to the desired isothermal crystallization temperature, i.e., 100 °C (3) and kept for 1 and 2 h at that temperature (4). Finally, the sample was cooled to 25 °C at 5 °C/min (5) and then heated to 200 °C at a heating rate of 10 °C/min (6). The second heating cycle was used to calculate the glass transition temperature (*T*_g_), cold crystallization temperature (*T*_cc_), and melting temperature (*T*_m_). The crystallinity (*X*_c_) of the PLA in the biocomposites was calculated using eq 1:
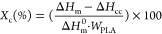
1where Δ*H*_m_ and Δ*H*_cc_ are the melting and cold crystallization enthalpies, respectively, and Δ*H*_m_^0^ is the enthalpy of fusion for 100% crystalline PLA (93 J/g).^8^*W*_PLA_ is the mass fraction of PLA in the composite samples.

### 3.5 Scanning Electron Microscopy (SEM)

To observe the internal morphology of the PLA and biocomposites, fractured under tensile testing, the surfaces were Au sputter coated utilizing a Denton vacuum sputter coater for 2 min at a 25% sputtering rate. The microstructure was then observed at different magnifications using a JEOL JSM 6390 microscope at an acceleration voltage of 5 kV. Neat BL samples were also tested, but the surface was not obtained under tensile testing due to the excessive brittleness of the Neat BL sample.

### 3.6 Mechanical Testing

The PLA_BL and PLA_BL-*g*-GMA biocomposites were tested under tension using an Instron 5966 (Norwood, MA, USA) with a 10 kN load cell. Type-V tensile specimens were tested at a controlled cross-head speed of 1 mm/min (ASTM D638 standard). At least four replicates in each composition were tested to obtain the mean and standard deviation of the tensile strength, Young’s modulus, and elongation at break.

## 4 Results and Discussion

### 4.1 FTIR Spectroscopy

**Figure 2:**
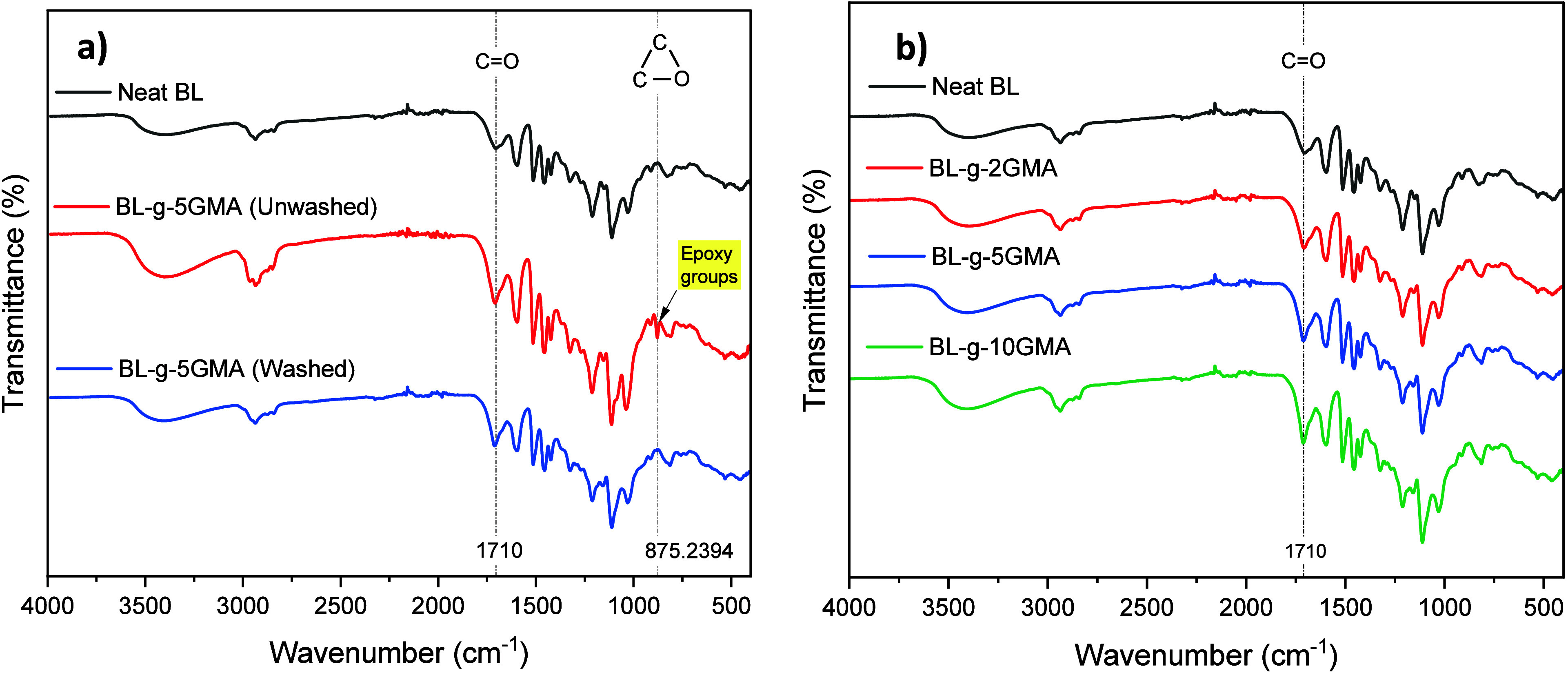
FTIR spectra of (a) neat BL, unwashed BL-*g*-5GMA, and washed BL-*g*-5GMA, and (b) neat BL and BL samples with 2, 5, and 10 wt % GMA.

The BL-*g*-5GMA samples were examined by FTIR to assess the grafting between GMA and BL. As it was anticipated that some unreacted GMA might remain present in the BL-*g*-5GMA samples after the melt mixing, FTIR analysis was performed not only on the as-processed BL-*g*-5GMA sample (unwashed) but also on the washed BL-*g*-5GMA sample. Figure 2a shows the FTIR spectra of unwashed BL-*g*-5GMA and washed BL-*g*-5GMA. The Neat BL sample, which is the as-received lignin without any modification, was also tested as the baseline, and its FTIR spectrum is given in Figure 2a. The BL spectra showed strong absorption bands at 3400 and 1710 cm^–1^ which belong to the −OH stretching vibration of phenolic hydroxyl groups and the C=O stretching of the carbonyl groups, respectively.^7,32^ The bands at 2935 and 2847 cm^–1^ were attributed to the C–H asymmetric and symmetric vibrations in the methyl and methylene groups. The BL characteristic peaks in the BL spectrum appeared at 1600 and 1516 cm^–1^, attributed to the vibration of the aromatic skeleton.^33^ The unwashed and washed BL-*g*-5GMA samples showed an increase in the peak’s intensity at 1710 cm^–1^, compared to Neat BL, which is attributed to the carbonyl groups of GMA. The unwashed BL-*g*-5GMA spectra showed a strong absorption band at around 875 cm^–1^, which is associated with the C–O–C stretching vibrations of the epoxy group in GMA. When GMA is not fully consumed in the grafting reaction with BL, some unreacted GMA molecules could remain in the mixture. The subsequent washing of the mixture with ethanol and distilled water led to the removal of these unreacted GMA molecules, which might explain the disappearance of the 875 cm^–1^ peak of the washed BL-*g*-5GMA sample. The absorption peaks at around 2950 cm^–1^ of all samples were attributed to the stretching vibration of C–H (CH_3_ and CH_2_). The presence of CH_3_ and CH_2_ was also confirmed by the absorption peaks at 1450 and 1380 cm^–1^, which were related with CH_3_ antisymmetric bending and deformation. The unwashed BL-*g*-5GMA spectra showed an increase in the peak intensity around 2950 cm^–1^. However, the peaks showed lower intensity in the washed samples.

The FTIR spectra of Neat BL and washed BL-*g*-GMA samples with 2, 5, and 10 wt % GMA are presented in Figure 2b. The characteristic vibrations of the carbonyl group at 1710 cm^–1^ increased with increasing amount of GMA, which could indicate an increase in the grafting yield. As the amount of GMA is increased in the mixture with lignin, more epoxy groups from GMA might have reacted with hydroxyl groups in lignin through an epoxy ring-opening reaction.^34^

In order to quantify the increase in the number of C=O chemical groups at the surface of the grafted lignin with increasing GMA content, the absorbance values of 1710 cm^–1^ and the percentage of increase in the C=O at 1710 cm^–1^ are extracted from Figure 2b and listed in Table 2. As shown in Table 2, the ungrafted Neat BL had the initial value of absorbance of 0.078 at 1710 cm^–1^, corresponding to the carbonyl group, C=O of BL. As the GMA content was increased from 0 to 10 wt %, the percentage of the C=O chemical group in BL-*g*-GMA samples increased proportionally. Compared to the Neat BL, there was about 51.7% increase in the C=O intensity with incorporating 10 wt % GMA. When the C=O peak height was normalized to that of C–H stretching vibrations (2935 cm^–1^), *A*_1710_/*A*_2935_ ratios of 1.40, 1.61, and 1.72 were obtained for BL-*g*-2GMA, BL-*g*-5GMA, and BL-*g*-10GMA, respectively, further supporting that the relative intensity of carbonyl group was increased with an increase in the GMA content.

**Table 2:** Intensity of the Absorbance Peak of 1710 cm^–1^ for BL-*g*-GMA Samples and Its Relative Increase with Respect to That of Neat BL

sample	GMA content (wt %)	*A*_1710_ (cm^–1^)	C=O increment at *A*_1710_ (%)
neat BL	0	0.087	
BL-*g*-2GMA	2	0.102	17.24
BL-*g*-5GMA	5	0.111	27.59
BL-*g*-10GMA	10	0.131	51.72

### 4.2 Rheological Properties

**Figure 3:**
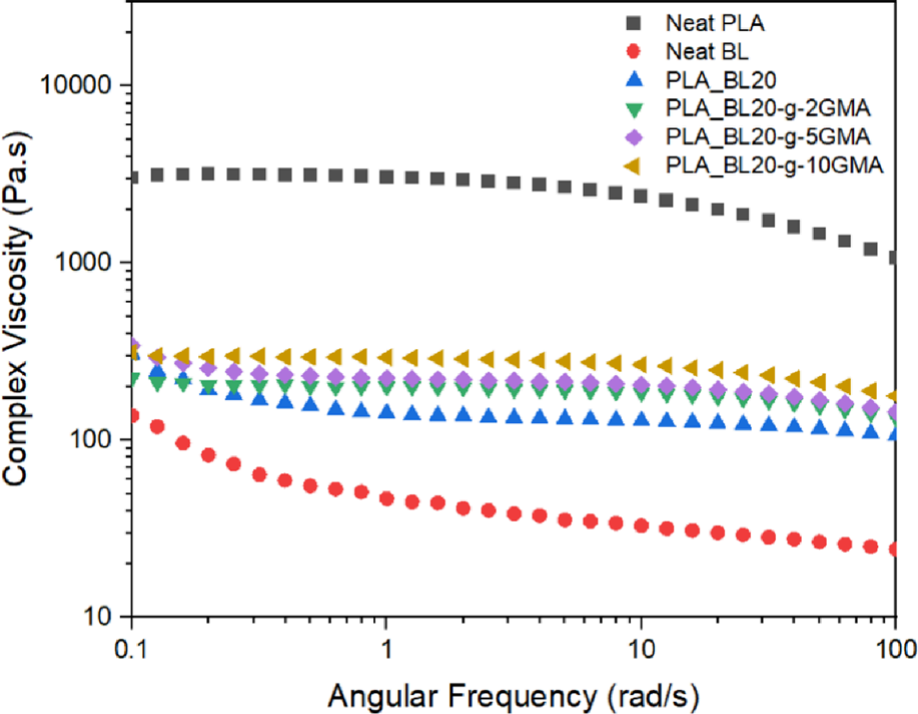
Complex viscosity (η*) at 170 °C as a function of frequency for PLA, BL, PLA_BL20, PLA_BL20-*g*-2GMA, PLA_BL20-*g*-5GMA, and PLA_BL20-*g*-10GMA biocomposites.

Figure 3 shows the complex viscosity as a function of frequency, measured at 170 °C, for Neat PLA, Neat BL, PLA_BL20, and PLA_BL20-grafted to 2, 5, and 10% GMA. It is observed that PLA exhibits a linear viscoelastic regime (LVR) at low frequencies, followed by a typical shear-thinning behavior of thermoplastics at higher frequencies. The shear-thinning behavior is caused by the PLA’s molecular chain disentanglements. The Neat BL sample showed non-Newtonian fluid behavior over the entire testing frequency range as its complex viscosity continuously decreased with an increase in frequency. The viscosity of BL was also significantly lower than that of PLA because BL has a relatively low molecular weight of 2.183 ± 40.5 g·mol^–1^, as reported in our previous study.^31^

Once 20 wt % BL was introduced to the PLA matrix, the PLA’s viscosity was reduced noticeably. The reason for this reduction is not only due to the lower viscosity of the BL phase, which is only 20 wt % of the blend but also because BL could have acted as a lubricant in the PLA matrix and reduced the PLA chain entanglement. The overall viscoelastic behavior of the PLA_BL20 sample was also similar to that of BL, as it did not exhibit linear and shear thinning regions.

However, the incorporation of BL-*g*-GMA in the PLA matrix caused an increase in the viscosity, compared to that of the PLA-BL20 composite. Moreover, the complex viscosity of these samples was further increased with an increase in the grafted amount of GMA from 2 to 10 wt % (Figure 3). Lignin-grafted GMA can promote cross-linking reactions with PLA due to the reaction of the acrylic groups in GMA with PLA and thus restrict the molecular mobility of PLA chains, which contributes to having a higher viscosity. Moreover, it is also plausible to assume that the unreacted GMA reacted with the hydroxyl and carboxyl end groups of PLA and further increased the viscosity.^28^ It is also interesting to note that PLA_BL20-*g*-GMA composites exhibited an overall viscoelastic behavior that was similar to that of Neat PLA. A plateau viscoelastic region was first observed at low frequencies, and the viscosity started to drop off toward the end of the test at high frequencies. The onset of shear thinning occurred at higher frequencies compared to Neat PLA. This is another indication of more difficult disentanglement of PLA molecules in the presence of BL20-*g*-GMA due to the reaction and cross-linking with GMA functional groups.

It is also expected that as the content of BL-grafted-GMA changes, the viscosity will be affected. On one hand, a higher content of lignin will tend to reduce the viscosity more, due to its inherently low viscosity. On the other hand, the addition of more BL-grafted-GMA will provide a larger number of functional groups that can react with PLA, which will increase the viscosity. Therefore, the exact viscosity at any given amount of BL-grafted-GMA will be controlled by these two competing factors.

### 4.3 Thermal Properties of PLA, BL, PLA_BL, and PLA_BL-*g*-GMA Composites

#### 4.3.1 Thermal Stability

**Figure 4:**
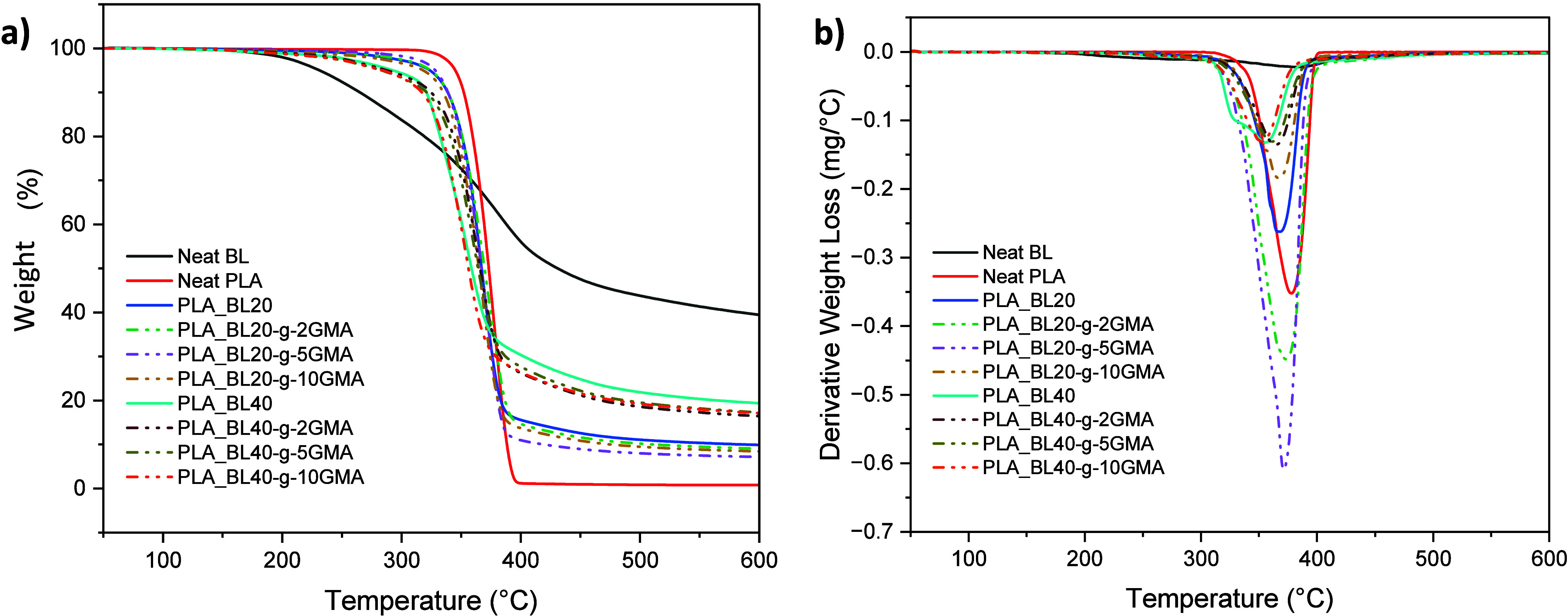
(a) TG and (b) DTG curves of Neat BL, Neat PLA, PLA_BL, and PLA_BL-*g*-GMA biocomposites.

The thermogravimetric (TG) and first derivative thermogravimetric (DTG) curves for Neat BL, Neat PLA, PLA_BL, and PLA_BL-*g*-GMA biocomposites are shown in Figure 4a,b, respectively. The TG curve of BL showed a very slow decomposition rate from about 262 to 429 °C, followed by a mass loss and a decrease in the rate. This indicates that the BL decomposition occurred during a multistage process, which is explained by the fact that lignin has various structural elements and functional groups that decompose at different temperatures.^35,36^ On the other hand, Neat PLA showed decomposition in a relatively narrow temperature range.

For Neat BL, Neat PLA, PLA_BL biocomposites, TGA data (*T*_5%,_*T*_onset_, *T*_max_, and the residual weight) are given in Table 3. An increase in the BL content led to an increase in the range of decomposition temperatures for PLA_BL biocomposites, which is reflected in the following sequence showing the temperatures corresponding to 5 wt % loss (*T*_5%_): PLA – 345 °C, PLA_BL20 – 324 °C, PLA_BL40 – 293 °C, as shown in Table 3. A high content of BL (40 wt %) also decreased the temperature at which the degradation of the biocomposites initiates, i.e., *T*_onset_, from 356 °C of Neat PLA to 322 °C of PLA_BL40. These reductions in both *T*_5%_ and *T*_onset_ can be explained by the fact that the start of decomposition of BL takes place at lower temperatures. The difference between *T*_5%_ and *T*_onset_ becomes larger as the BL content is further increased in the biocomposites. Comparing Neat PLA and PLA_BL40, this difference increases by about 6.8%, which indicates that an increase in the content of BL leads to destabilizing effects on PLA and speeds up its thermal degradation. The calculated BL residue at 600 °C was about 39.47%, which can be explained by the formation of certain aromatic structures (highly condensed) that have the ability to form char.^37^ As informed by Brebu et al.,^36^ unstable free radicals can be formed at high temperatures due to the cleavage of aryl–ether linkages. Since these radicals are highly reactive, they can further react chemically by radical interactions, rearrangement, or electron abstraction and form more stable products. An increase in PLA_BL residue was observed from 0.81 wt % for Neat PLA to 19.38 wt % for PLA_BL40. Also, the maximum decomposition temperature (*T*_max_) decreased with increasing the amount of BL, from 378 °C of Neat PLA to 356 °C of PLA_BL40. Analyzing the DTG graph of PLA_BL40 revealed two peaks that were caused by the high content of BL and the different decomposition processes of PLA and lignin.

**Table 3:** TGA Data for Neat BL, Neat PLA, PLA_BL, and PLA_BL-*g*-GMA Biocomposites

composite	*T*_5%_ (°C)	*T*_onset_ (°C)	*T*_max_ (°C)	residue (%)
neat BL	231.00	262.30		39.47
neat PLA	345.00	356.05	378.00	0.81
PLA_BL10	338.00	352.62	379.67	4.92
PLA_BL10-*g*-2GMA	335.00	353.33	382.00	5.36
PLA_BL10-*g*-5GMA	337.67	354.96	385.00	4.30
PLA_BL10-*g*-10GMA	315.33	334.69	357.33	5.90
PLA_BL20	323.67	346.73	368.33	9.90
PLA_BL20-*g*-2GMA	324.33	334.50	373.33	9.04
PLA_BL20-*g*-5GMA	328.00	347.26	371.33	7.17
PLA_BL20-*g*-10GMA	318.00	342.86	367.33	8.43
PLA_BL30	311.33	339.94	362.67	13.56
PLA_BL30-*g*-2GMA	309.67	344.03	371.00	12.06
PLA_BL30-*g*-5GMA	312.33	343.08	369.33	10.33
PLA_BL30-*g*-10GMA	298.67	339.74	365.67	12.46
PLA_BL40	292.67	321.99	355.67	19.38
PLA_BL40-*g*-2GMA	287.33	337.55	364.67	16.43
PLA_BL40-*g*-5GMA	283.00	334.40	360.67	17.27
PLA_BL40-*g*-10GMA	284.00	326.83	353.00	17.11

Furthermore, as seen in Table 3, PLA composites containing 10, 20, and 30 wt % modified BL (BL-*g*-GMA) exhibited a small increase in *T*_5%_ when the GMA content was increased from 2 to 5 wt %. This could be due to the increased reaction between GMA and PLA that enhanced the material’s thermal stability. However, with further increase of the GMA content from 5 to 10 wt % caused a reduction in *T*_5%_, and the composites having 10, 20, and 30 wt % BL-*g*-10GMA showed a reduction in *T*_5%_ of 6.7, 1.8, and 4.0%, respectively, compared to their respective BL counterparts. This was probably due to the presence of a great amount of unreacted GMA at high GMA loadings, which decomposed at lower temperatures. Abdelwahab et al.^38^ observed a significant reduction in thermal degradation when 16.8 wt % GMA was incorporated into PLA composite with 21 wt % organosolv lignin and related it to the destabilizing effect of GMA on the composite.

Overall, as the content of BL-*g*-GMA increased from 10 to 40 wt %, the residue also increased, with a trend similar to that of the unmodified BL case (Table 3). However, except for the case of 10 wt % lignin, the residue was measured to be smaller for the BL-*g*-MA-containing samples compared to BL-containing samples. For instance, the residue values were 19.4 and 17.1% in the composites with 40 wt % BL and 40 wt % BL-*g*-GMA, respectively. It is also interesting to note that at a given lignin content (10–30 wt %), the residue decreased with an increase in the GMA content from 2 to 5 wt % and increased by further increasing the GMA content to 10 wt %. A similar optimal trend was also observed for *T*_5%_, suggesting that 5 wt % GMA might be an optimal loading. It is believed that these changes in the residue are governed by a balance between reacted and unreacted GMA. While reacted GMA promotes thermal stability and lowers the residue, unreacted GMA can decompose at lower temperatures with minimal residue, causing a reduction in thermal stability as well as total residue.

#### 4.3.2 Softening, Melting and Crystallization

**Figure 5:**
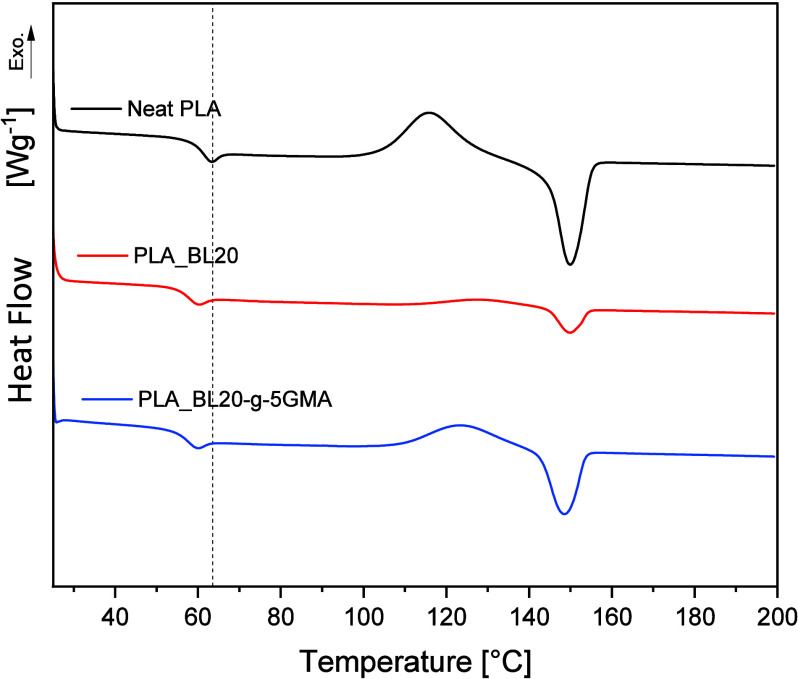
DSC thermograms of 2nd heating scans (after cooling at a rate of 5 °C/min) of Neat PLA and PLA_BL20 and PLA_BL20-*g*-5GMA.

The second heating thermograms of Neat PLA, PLA_BL20, and PLA_BL20-*g*-5GMA are presented in Figure 5. By analyzing these thermograms, the glass transition temperatures (*T*_g_), cold crystallization temperatures (*T*_cc_), cold crystallization enthalpy (Δ*H*_cc_), melt temperature (*T*_m_), and melting enthalpy (Δ*H*_cc_) were identified, and the crystallinity values (*X*_c_) are calculated and summarized in Table 4. The *T*_g_ of PLA decreased from 58.8 to 54.9 °C after the incorporation of 20 wt % BL. This reduction in the *T*_g_ with the addition of BL was related to the increase in chain mobility in PLA_BL blends.^39^ The introduction of 5 wt % GMA did not appear to have a significant effect on the *T*_g_. This could be due to having two competing factors of reduced molecular mobility because of reacted GMA and the plasticizing effect of unreacted GMA. Abdelwahab et al.^36^ reported that the addition of GMA into PLA/organosolv lignin decreased the *T*_g_ significantly, from 62 °C in Neat PLA to 39 °C in PLA with 21 wt % organosolv lignin with 16.8 wt % GMA. According to these authors, GMA acted as a toughening agent and increased the polymer chain mobility.

**Table 4:** Glass Transition Temperature (*T*_g_), Cold Crystallization Temperature (*T*_cc_), Cold Crystallization Enthalpy (Δ*H*_cc_), Melt Temperature (*T*_m_), Melting Enthalpy (Δ*H*_m_), and Crystallinity (*X*_c_) of Neat PLA, PLA_BL20, and PLA_BL20-*g*-5GMA, Obtained from the DSC 2nd Heating Cycles

composite	*T*_g_ (°C)	*T*_cc_ (°C)	Δ*H*_cc_ (J/g)	*T*_m_ (°C)	Δ*H*_m_ (J/g)	*X*_c_ (%)
neat PLA	58.27	104.00	22.53	144.29	24.06	1.64
PLA_BL20	54.93	111.68	5.43	144.48	5.89	0.49
PLA_BL20-*g*-5GMA	54.18	108.00	13.16	143.00	13.88	0.77

The DSC thermogram of Neat PLA in the second heating cycle appeared with a distinct exothermic curve starting at 104 °C and peaking at 116 °C, as shown in Figure 5. Nevertheless, after cooling the sample at a rate of 5 °C/min, the crystallinity of Neat PLA, calculated from the enthalpies, showed a low percentage of 1.64%. Compared to the Neat PLA, PLA_BL20 composites had an increase in *T*_cc_ to 112 °C and a decrease in crystallinity degree to 0.49%. The cold crystallization enthalpy (Δ*H*_cc_) also decreased from 22.53 J/g in Neat PLA to 5.43 J/g in the composite with 20 wt % BL (Table 4). This inferior crystallization behavior may be attributed to the difficulty of PLA chain stacking. In other words, the proper ordering and stacking of PLA chains were delayed or hindered in the presence of a high amount of amorphous BL. Gordobil et al.^8^ reported that the introduction of lignin, which is amorphous in nature, influences the interactions among the PLA chains during crystallization. However, the incorporation of BL-*g*-GMA into the PLA matrix instead of the unmodified BL caused an increase in both Δ*H*_cc_ and *X*_c_ as well as a reduction in *T*_cc_ (Table 4). Even though the change in *X*_c_ was insignificant, these findings suggest that BL-*g*-GMA favored the PLA’s crystallinity more than just BL.

#### 4.3.3 Effect of the Cooling Rate on the Thermal Behavior

**Figure 6:**
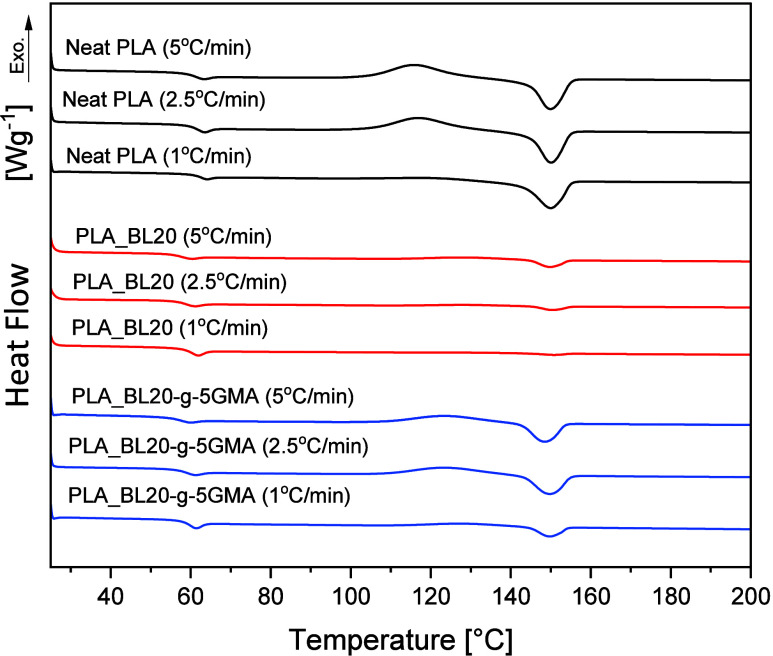
DSC thermograms of 2nd heating scans of Neat PLA and PLA_BL20 and PLA_BL20-*g*-5GMA after cooling at different rates.

To further explore the crystallization behavior of the biocomposites, the influence of cooling rate (5, 2.5, and 1 °C/min) on the thermal behavior of Neat PLA, PLA_BL20, and PLA_BL20-*g*-5GMA was also investigated, and the corresponding thermograms and results are presented in Figure 6 and Table 5. As expected, the *X*_c_ of PLA increased with a decrease in the cooling rate. It increased from 1.64% at a cooling rate of 5 °C/min to 23.97% at a cooling rate of 1 °C/min, which is related to the low crystallization rate of PLA.^40^ In other words, PLA requires relatively longer times to obtain an ordered arrangement of its molecular chains. Faster cooling rates restrict the time during which the PLA chains can be ordered, whereas slower cooling rates provide longer times for the chains to move and arrange themselves more effectively in the crystalline structure. The *T*_g_ of PLA also increased with a decrease in the cooling rate. Slower cooling rates tend to raise *T*_g_ which is probably because slower cooling rates allow molecular chains to align and pack more densely, further reducing the free volume. However, the value of *T*_g_ generally depends on various factors such as molecular weight, intermolecular interaction, chain mobility, and the presence of a crystalline phase.^41,42^ It is also noted that *T*_cc_ of Neat PLA decreased as the cooling rate was lowered from 5 to 1 °C/min, indicating that the PLA samples cooled with a lower cooling rate crystallized more easily during the heating process. In PLA_BL20 and PLA_BL20-*g*-5GMA, the changes in *T*_g_ and Δ*H*_cc_ as a function of cooling rate followed the behaviors observed for Neat PLA. However, Δ*H*_m_ decreased quite significantly with a decrease in the cooling rate, resulting in an insignificant change in *X*_c_, such that *X*_c_ remained under 2% for all the cooling rates for all of the lignin-containing samples. In other words, cooling rates as low as 1 °C/min were insufficient to introduce any appreciable crystallinity on the samples containing lignin.

**Table 5:** Glass Transition Temperature (*T*_g_), Cold Crystallization Temperature (*T*_cc_), Melt Temperature (*T*_m_), and Crystallinity (*X*_c_) of Neat PLA, PLA_BL20, and PLA_BL20-*g*-5GMA, Obtained from the DSC 2nd Heating Cycle after Cooling with Different Rates

composite	cooling rate (°C/min)	*T*_g_ (°C)	*T*_cc_ (°C)	Δ*H*_cc_ (J/g)	*T*_m_ (°C)	Δ*H*_m_ (J/g)	*X*_c_ (%)
neat PLA	5	58.27	104.00	22.53	144.29	24.06	1.64
	2.5	59.07	104.65	21.14	144.43	24.07	3.13
	1	60.19	100.42	2.67	142.33	25.98	23.97
PLA_BL20	5	54.93	111.68	5.43	144.48	5.89	0.49
	2.5	56.04	112.00	3.89	144.71	4.26	0.39
	1	57.82	112.49	1.28	145.12	1.79	0.54
PLA_BL20-*g*-5GMA	5	54.18	108.54	13.16	142.59	13.88	0.77
	2.5	55.95	108.60	13.78	143.17	14.80	1.10
	1	57.43	112.81	5.04	144.58	6.15	1.19

#### 4.3.4 Isothermal Crystallization of PLA, PLA_BL20, and PLA_BL20-*g*-5GMA

**Figure 7:**
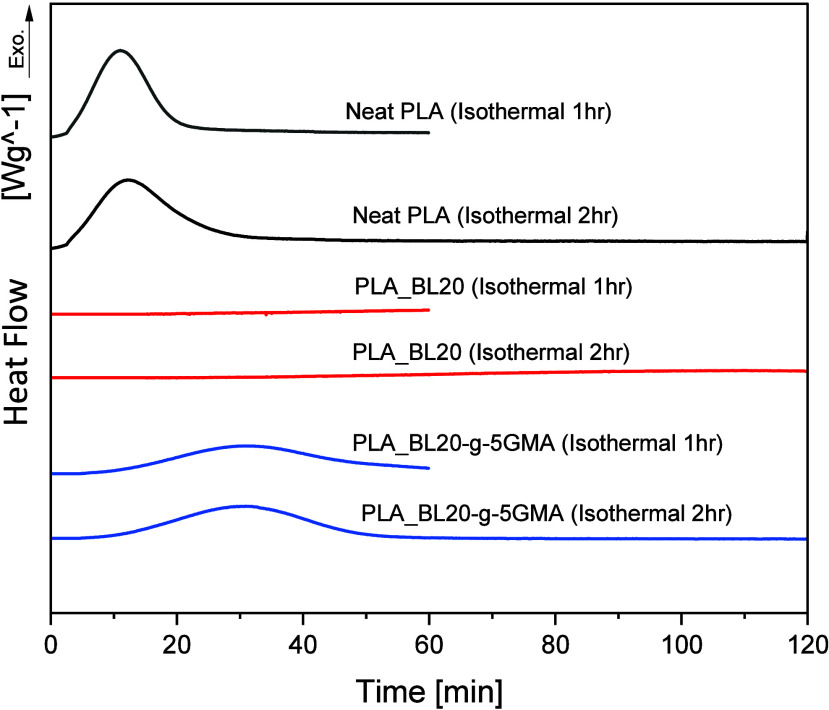
DSC thermograms of the isothermal crystallization at 100 °C.

As PLA_BL20 and PLA_BL20-*g*-5GMA showed low crystallinity at even a low cooling rate (1 °C/min), while PLA exhibited a significant increase in crystallinity at the same cooling rate, it was also worth investigating the isothermal crystallization behavior of the composites. The isothermal crystallization results of the processed Neat PLA, PLA_BL20, and PLA_BL20-*g*-5GMA for 1 and 2 h at 100 °C are shown in Figure 7, and the results are summarized in Table 6. As seen in Figure 7, the crystallization kinetics of PLA resulted in a relatively rapid initial crystallization in the first 20 min, followed by a plateau or a very slow increase in crystallinity. This explains why a longer isothermal heating time of 2 h did not significantly increase the crystallinity. The *X*_c_ of PLA with 1 h isothermal heating was 27.86%, which is slightly higher than the nonisothermal cases (*X*_max_ = 23.97% at 1 °C/min). The addition of unmodified BL to the PLA matrix decelerated the crystallization kinetics and resulted in much lower *X*_c_ (3.15%), even with 2 h isothermal heating. This value was, however, slightly higher, compared to *X*_c_=0.54% at 1 °C/min cooling rate (nonisothermal) case. These observations suggest that the crystallization rate at 100 °C for PLA_BL20 is extremely slow. Various factors affect the crystallinity of PLA in the presence of lignin, including the interactions between PLA and lignin, lignin content, and lignin molecular weight. This indicates that the poor dispersion and high loadings of BL (20 wt %) disrupted the regular arrangement of PLA chains during the isothermal process. Ye et al.^43^ reported that adding lignosulfonate lignin to PLA can reduce its crystallinity, and the lignin content and PLA-lignin interactions determine the degree of reduction.

**Table 6:** Crystallization Enthalpy (Δ*H*_c_) and Crystallinity (*X*_c_) of PLA, PLA_BL20, and PLA_BL20-*g*-5GMA at 100 °C Isothermal Temperature for 1 and 2 h

composite	isothermal time (h)	Δ*H*_c_ (J/g)	*X*_c_ (%)
neat PLA	1	25.94	27.86
	2	28.55	30.66
PLA_BL20	1	2.06	2.21
	2	2.93	3.15
PLA_BL20-*g*-5GMA	1	21.12	22.78
	2	24.41	26.22

It is interesting to observe that the PLA_BL20-*g*-5GMA with 2 h isothermal crystallization showed an *X*_c_ value comparable to Neat PLA but with a slower crystallization rate (Figure 7). The *X*_c_ of PLA_BL20-*g*-5GMA increased significantly from 1.19% at 1 °C/min cooling rate to 22.78 and 26.22% at 1 and 2 h isothermal heating, respectively. This difference between BL20 and BL20-*g*-5GMA could be related to the presence of both reacted and unreacted GMA. The epoxy groups of GMA react with the hydroxyl groups in lignin to reduce the surface polarity of lignin and improve the interfacial compatibility. As discussed in the next section (Section 4.4), this results in a better dispersion of lignin particles in PLA matrix with smaller sizes and greater numbers and therefore provides a stronger crystal nucleating ability. Moreover, the presence of unreacted GMA can enhance the PLA chain mobility, which can enhance crystal growth.

### 4.4 Morphological Analysis

**Figure 8:**
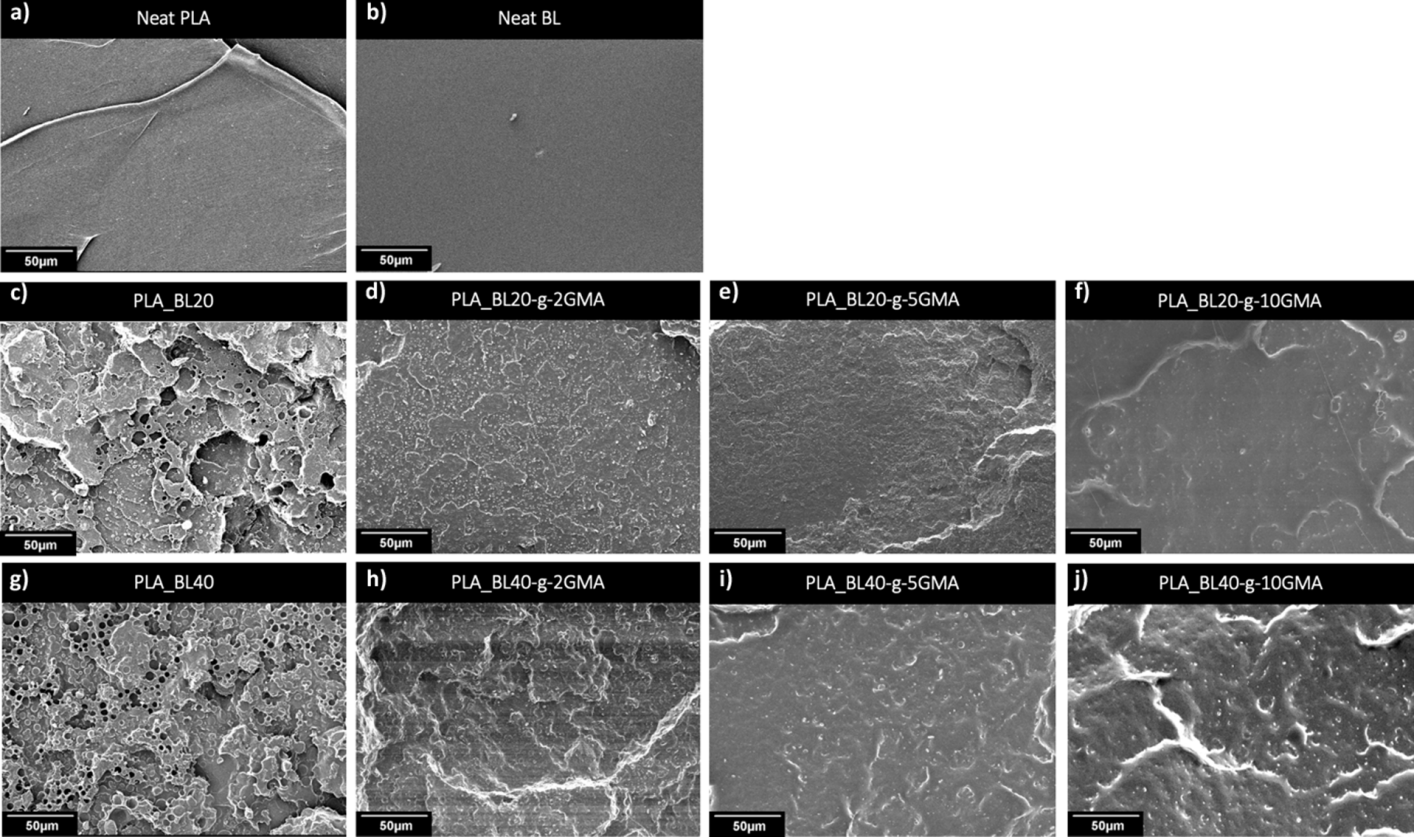
SEM micrographs of (a) tensile fractured surface of Neat PLA, (b) fractured surface of extruded BL at 120 °C, and tensile fractured surface of (c) PLA_BL20, (d) PLA_BL20-*g*-2GMA, (e) PLA_BL20-*g*-5GMA, (f) PLA_BL20-*g*-10GMA, (g) PLA_BL40, (h) PLA_BL40-*g*-2GMA, (i) PLA_BL40-*g*-5GMA, and (j) PLA_BL40-*g*-10GMA.

Figure 8a,b shows the SEM micrographs of the fractured surfaces of Neat PLA and Neat BL (processed at 120 °C), whereas Figure 8c–j shows PLA_BL20, PLA_BL20-*g*-(2, 5, and 10 wt % GMA), PLA_BL40, and PLA_BL40-*g*-(2,5 and 10 wt % GMA). The micrograph of the fractured surface of Neat PLA (Figure 8a) and Neat BL (Figure 8b) demonstrates a brittle structure with a smooth surface. As seen in Figure 8c(PLA_BL20) and Figure 8g(PLA_BL40), the introduction of unmodified BL at 20 and 40 wt % resulted in relatively large BL particle sizes where a clear separation between the PLA matrix and BL particles is visible on the fracture surface, indicating a poor interfacial adhesion and the dominance of interface separation as the failure mechanism. Also, as the BL content was increased from 20 to 40 wt %, the lignin particle size was increased from 3.3 to 4.4 μm due to BL aggregation, driven by the interaction of hydrogen bonds among carbonyl, phenolic, and aliphatic hydroxyl groups. With increasing concentrations of BL, adhesion between BL particles and the PLA matrix further decreased, indicating limited miscibility between BL and PLA.

**Figure 9:**
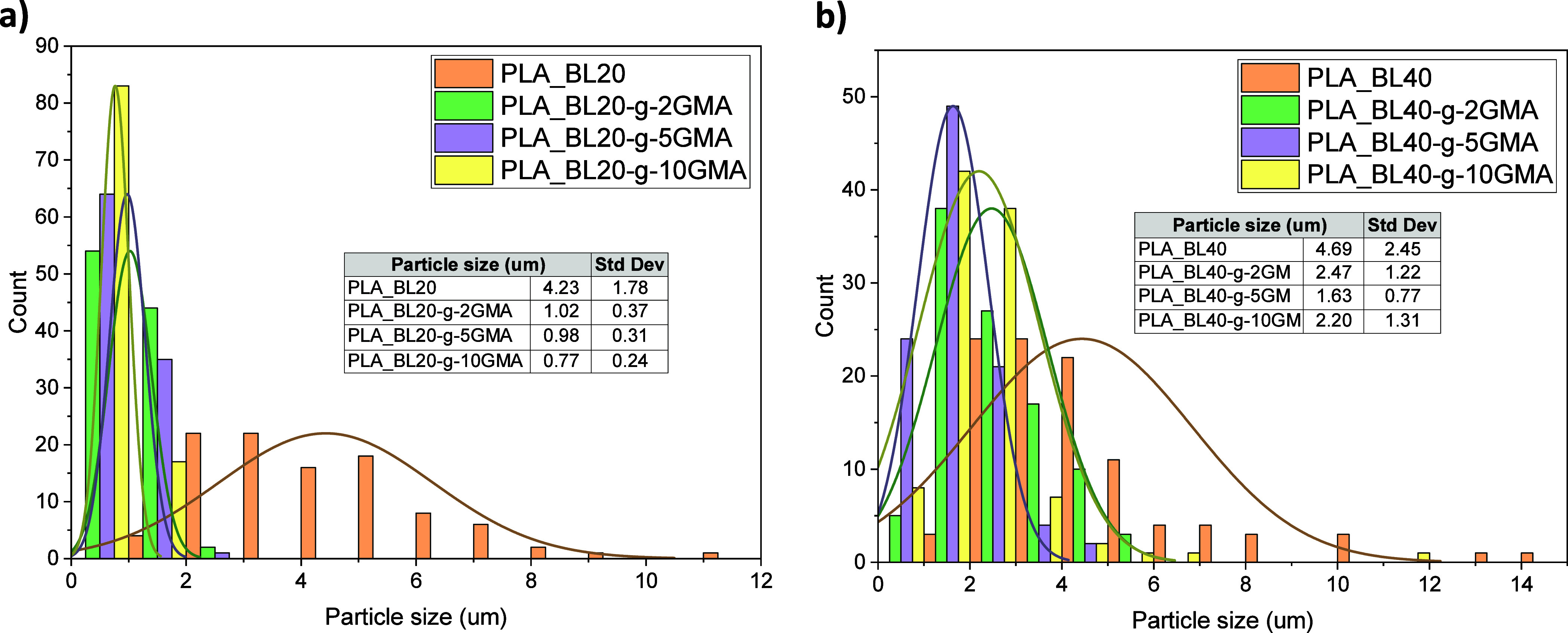
Particle size distribution histogram determined from the SEM micrographs for (a) PLA_BL20 and PLA_BL20-*g*-2,5 and 10GMA, (b) PLA_BL40 and PLA_BL40-*g*-2,5 and 10GMA.

Comparing the composites in Figure 8c through 8j reveals that the use of BL-*g*-GMA instead of Neat BL significantly improved the miscibility of lignin in the PLA matrix in all the composites with different BL contents of 20 and 40 wt % as well as different ratios of grafted GMA of 2, 5, and 10 wt %. This resulted in a more uniform distribution of BL domains in the PLA matrix as well as a considerable decrease in the BL domain size, as the corresponding histograms of the BL particle size distribution demonstrate (Figure 9). The domain size decreased from 4.2 μm in PLA_BL20 to 0.8 μm in PLA_BL20-*g*-10GMA, which was an around 82% reduction. It is also noted that the domain size was continuously reduced as the GMA content was increased. In the composites with 40 wt % BL, the domain size also decreased, but it showed a lower reduction, from 4.7 to 1.6 μm accounting for about 65% reduction. It is also seen that the minimum domain size was obtained at 5 wt % GMA instead of 10 wt %, which might suggest that there is an optimum GMA content for a given PLA and lignin ratio. One potential reason for the smaller size reduction could be related to the very high loading of lignin in this case (40 wt %). Moreover, the voids almost disappeared from the morphology of the samples containing GMA, indicating an improved interfacial interaction between PLA and grafted BL. In addition, the overall fracture surface of PLA_BL-*g*-GMA composites exhibited a less brittle characteristic, as is evident by the presence of wrinkles on their morphologies, which are distinctly different from those of PLA_BL samples. One reason for this difference could be the presence of residual unreacted GMA acting as a plasticizer.

### 4.5 Mechanical Properties

**Figure 10:**
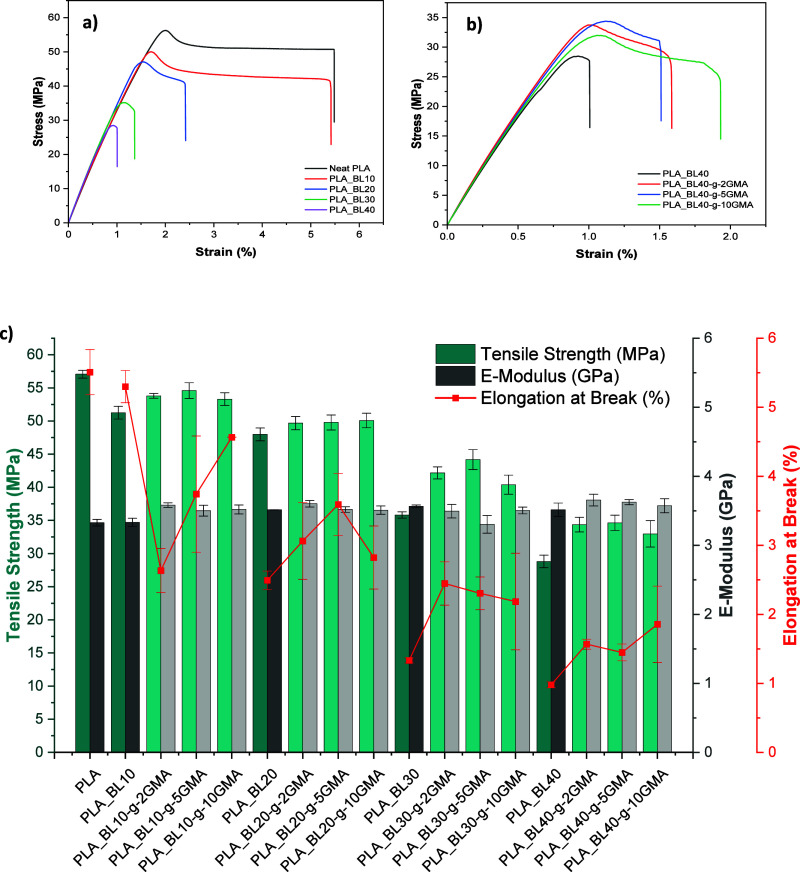
Tensile stress–strain plots of (a) PLA_BL composites, and (b) PLA_BL40-*g*-0, 2, 5, and 10 GMA and (c) tensile strength, Young’s modulus, and elongation at break of Neat PLA and PLA_BL and PLA_BL-*g*-GMA biocomposites.

Figure 10a,b shows the tensile stress–strain plots of PLA_BL and PLA_BL40-*g*-GMA biocomposites, respectively. Figure 10c and Table 7 also present the tensile strength values, Young’s modulus, and elongation at break for all the biocomposites. As expected, there is a consistent drop in the strength and elongation at break with an increase in the unmodified BL content (Figure 10a), which was ascribed to the lower strength and higher rigidity of BL compared to PLA, as well as the aggregation of BL and low interface interaction between PLA and BL.

**Table 7:** Results of Tensile Measurements of Neat PLA and PLA_BL and PLA_BL-*g*-GMA Biocomposites^a^

composite	TS		*E*		EB	
	MPa	std.	GPa	std.	%	std.
PLA	57.08	0.58	3.33	0.05	5.51	0.33
PLA_BL10	51.26	0.96	3.33	0.06	5.30	0.23
PLA_BL10-*g*-2GMA	53.80	0.37	3.58	0.03	2.63	0.32
PLA_BL10-*g*-5GMA	54.59	1.188	3.50	0.08	3.74	0.84
PLA_BL10-*g*-10GMA	53.31	0.97	3.52	0.07	4.57	0.00
PLA_BL20	48.00	0.96	3.51	0.00	2.49	0.13
PLA_BL20-*g*-2GMA	49.69	1.00	3.60	0.05	3.06	0.56
PLA_BL20-*g*-5GMA	49.79	1.14	3.52	0.04	3.59	0.45
PLA_BL20-*g*-10GMA	50.08	1.10	3.51	0.06	2.82	0.46
PLA_BL30	35.83	0.48	3.57	0.02	1.33	0.04
PLA_BL30-*g*-2GMA	42.17	0.91	3.49	0.10	2.45	0.31
PLA_BL30-*g*-5GMA	44.19	1.50	3.30	0.13	2.30	0.24
PLA_BL30-*g*-10GMA	40.40	1.44	3.50	0.05	2.19	0.70
PLA_BL40	28.81	0.95	3.51	0.10	0.98	0.04
PLA_BL40-*g*-2GMA	34.36	1.10	3.66	0.08	1.57	0.07
PLA_BL40-*g*-5GMA	34.63	1.17	3.62	0.04	1.45	0.12
PLA_BL40-*g*-10GMA	32.97	1.99	3.57	0.10	1.85	0.55

a TS, *E,* and EB are tensile strength, Young’s modulus, and elongation at break, respectively.

The incorporation of BL-*g*-GMA to PLA, instead of unmodified BL, improved the tensile strength in all the cases and increased the elongation at break at high lignin loadings of 30 and 40 wt %. Yong’s modulus did not vary significantly. These improvements were attributed to the reactions between PLA and BL-*g*-GMA as well as the improvements in the dispersion and distribution of lignin in PLA (Figure 8c–j). The reaction between the GMA of BL-*g*-GMA and the PLA matrix enhanced the load transfer capabilities at the interface of the two components. Composites with 30 and 40 wt % BL-*g*-GMA BL showed greater improvements in the tensile strength compared to those with less amount of modified BL, which could be related to the fact that the GMA content increased with an increase in the lignin content. For instance, the PLA_BL30-*g*-5GMA biocomposite showed the highest improvement of 23% in the tensile strength, increasing from 35.8 MPa for PLA_BL30 to 44.2 MPa.

The elongation at the break of PLA_BL10-*g*-2GMA was significantly lower than that of PLA_BL10, accounting for about 50.1% reduction. This reduction might be due to insufficient GMA, which was only 0.2 wt % of total composite mass, and perhaps left no unreacted GMA behind to act as a toughener. As a result of the high interaction between PLA and BL-*g*-GMA with no unreacted GMA, Young’s modulus in this sample increased and showed the highest improvement of 7.5%. However, a smaller reduction of 29.4 and 13.8% was observed in the same composites containing greater amounts of GMA (5 and 10 wt % of BL’s weight), which also resulted in lower improvement in the modulus, i.e., 5.1 and 5.6%, respectively. This might be due to the increase in the unreacted GMA amount in these composites.

Furthermore, the elongation at break showed a substantial increase in the composites containing a higher percentage of modified BL (i.e., 20, 30, and 40 wt %) due to the higher amount of unreacted GMA. The greatest enhancement was about 90% for the highest lignin and GMA contents, i.e., PLA_BL40-*g*-10GMA. Even though the modulus was also affected by lignin modification, its dependency was not as significant as that of strength and elongation at break. The modulus appeared to increase at very low and very high lignin contents (i.e., 10 and 40) but decrease at mediocre lignin content (i.e., 30 wt %), which the counteracting impacts of reacted and unreacted GMA could explain.

As the ratio of PLA and GMA varies for composites having different ratios of lignin, the influence on the mechanical properties also varies from one composition to another. For instance, the composite with 40 wt % modified BL showed an exception, increasing all mechanical properties (tensile strength, Young’s modulus, and elongation at break), as seen in Figure 10b and Table 7. PLA_BL40-*g*-10GMA increased around 90% in elongation at break; however, the tensile strength and Young’s modulus improved less than other composites with 40 wt % modified BL.

## 5 Conclusions

In this study, bioleum (BL) was successfully grafted with glycidyl methacrylate (GMA) using a melt mixing process. The obtained BL-grafted-GMA was then used to blend with PLA via the melt extrusion method in the presence of a small amount of dicumyl peroxide (DCP) that served as a free radical initiator. FTIR results indicated an increase in the absorbance intensity of the carbonyl group band in BL/GMA mixtures, confirming the grafting of GMA and lignin. The incorporation of BL-*g*-GMA in the PLA matrix provided a higher complex viscosity than that of the composites with PLA_BL. The complex viscosity of PLA_BL-*g*-GMA samples showed a shear thinning behavior and increased with an increase in the GMA content. The crystallinity of PLA was hindered by BL and BL-*g*-GMA even with cooling rates reduced from 5 to 1 °C. However, it was observed that the composites with BL-*g*-GMA displayed crystallinity values comparable to those of PLA during isothermal crystallization but at a slower rate. SEM analysis demonstrated that using BL-*g*-GMA instead of Neat BL provided far better dispersion and compatibility of lignin in the PLA matrix, resulting in reduced BL domain size, reduced voids, and a more uniform distribution of BL in the PLA matrix. All composites with BL-*g*-GMA showed improvement in tensile strength compared with the composites with Neat BL. The PLA_BL30-*g*-5GMA biocomposite exhibited the highest improvement of 23% in the tensile strength. Moreover, the biocomposites with the highest lignin loading (40 wt %) enhanced all the tensile properties once GMA was incorporated. The results of this work demonstrate lignin-GMA grafting as an efficient and easily scalable method to prepare high-performance biocomposites at high lignin loadings.

## Data Availability

Data is provided within the manuscript figures and tables.

## Notes

While preparing this work, the authors did not use any AI tool/service to generate any portion of the writing/content for this paper.
